# Adipose-Derived Mesenchymal Stem Cells From a Hypoxic Culture Improve Neuronal Differentiation and Nerve Repair

**DOI:** 10.3389/fcell.2021.658099

**Published:** 2021-04-30

**Authors:** Szu-Hsien Wu, Yu-Ting Liao, Kuang-Kai Hsueh, Hui-Kuang Huang, Tung-Ming Chen, En-Rung Chiang, Shan-hui Hsu, Ting-Chen Tseng, Jung-Pan Wang

**Affiliations:** ^1^Division of Plastic and Reconstructive Surgery, Department of Surgery, Taipei Veterans General Hospital, Taipei, Taiwan; ^2^Department of Surgery, School of Medicine, National Yang Ming Chiao Tung University, Taipei, Taiwan; ^3^Department of Orthopaedics and Traumatology, Taipei Veterans General Hospital, Taipei, Taiwan; ^4^Department of Orthopedics, Taoyuan General Hospital, Ministry of Health and Welfare, Taoyuan, Taiwan; ^5^Department of Orthopaedics, Ditmanson Medical Foundation, Chiayi Christian Hospital, Chiayi, Taiwan; ^6^Department of Food Nutrition, Chung Hwa University of Medical Technology, Tainan, Taiwan; ^7^Division of Orthopedics, Taipei City Hospital-Zhong Xiao Branch, Taipei, Taiwan; ^8^Institute of Polymer Science and Engineering, National Taiwan University, Taipei, Taiwan

**Keywords:** ADSC, BMSC, hypoxic culture, neuronal differentiation, nerve repair

## Abstract

Hypoxic expansion has been demonstrated to enhance *in vitro* neuronal differentiation of bone-marrow derived mesenchymal stem cells (BMSCs). Whether adipose-derived mesenchymal stem cells (ADSCs) increase their neuronal differentiation potential following hypoxic expansion has been examined in the study. Real-time quantitative reverse transcription-polymerase chain reaction and immunofluorescence staining were employed to detect the expression of neuronal markers and compare the differentiation efficiency of hypoxic and normoxic ADSCs. A sciatic nerve injury animal model was used to analyze the gastrocnemius muscle weights as the outcomes of hypoxic and normoxic ADSC treatments, and sections of the regenerated nerve fibers taken from the conduits were analyzed by histological staining and immunohistochemical staining. Comparisons of the treatment effects of ADSCs and BMSCs following hypoxic expansion were also conducted *in vitro* and *in vivo*. Hypoxic expansion prior to the differentiation procedure promoted the expression of the neuronal markers in ADSC differentiated neuron-like cells. Moreover, the conduit connecting the sciatic nerve gap injected with hypoxic ADSCs showed the highest recovery rate of the gastrocnemius muscle weights in the animal model, suggesting a conceivable treatment for hypoxic ADSCs. The percentages of the regenerated myelinated fibers from the hypoxic ADSCs detected by toluidine blue staining and myelin basic protein (MBP) immunostaining were higher than those of the normoxic ones. On the other hand, hypoxic expansion increased the neuronal differentiation potential of ADSCs compared with that of the hypoxic BMSCs *in vitro*. The outcomes of animals treated with hypoxic ADSCs and hypoxic BMSCs showed similar results, confirming that hypoxic expansion enhances the neuronal differentiation potential of ADSCs *in vitro* and improves *in vivo* therapeutic potential.

## Introduction

Nerve grafts, which are composed of natural or artificial tubes, are considered ideal treatments to guide nerve regeneration across the injury gaps (Faroni et al., 2013, 2014). Artificial conduits with bone marrow-derived mesenchymal stem cells (BMSCs) have been found to benefit peripheral nerve regeneration (Frattini et al., 2012; Wang et al., 2020). The advantages of mesenchymal stem cells in facilitating neurogenesis might be due to varied neurotrophic factor secretion (Uccelli et al., 2011). As adipose-derived mesenchymal stem cells (ADSCs) can be easily collected from subcutaneous fat tissue by liposuction without great pain, more cells can be obtained (Gimble et al., 2007). Furthermore, ADSCs show no significant differences in differentiation capacity, cell senescence, and efficiency of gene transduction compared with BMSCs (De Ugarte et al., 2003; Fernandes et al., 2018).

Utilization of ADSCs to treat and improve nerve regeneration in animal models with peripheral nerve defects has been well reported (Di Summa et al., 2010; Erba et al., 2010; Liu et al., 2011; Scholz et al., 2011; Marconi et al., 2012; Orbay et al., 2012; Sowa et al., 2016). ADSCs can differentiate into Schwann cells (SCs)-like (Kingham et al., 2007; Di Summa et al., 2014) and neuron-like (Scholz et al., 2011; New et al., 2015) phenotypes *in vitro* and *in vivo* to promote nerve regeneration. In addition, ADSCs secrete neurotrophic factors such as vascular endothelial growth factors (VEGFs), brain-derived growth factors (BDNFs), nerve growth factors (NGFs), and glial line-derived neurotrophic factors (GDNFs) to facilitate intrinsic healing utilizing host inhabited SCs (Widgerow et al., 2013; Faroni et al., 2014). The neuroregenerative effect of ADSCs has been found to be comparable to transplantation of SCs (Sowa et al., 2016). SCs are difficult to harvest and expand (Kocsis et al., 2002), and the harvesting procedure might cause donor-site morbidity. In addition, the de-differentiation, macrophage recruitment and myelin clearance functions of SCs are impaired in elderly hosts (Painter et al., 2014). Therefore, ADSCs can serve as an alternative clinical application. Intravenous injections of ADSCs have been used to treat multiple nerve injury sites (Marconi et al., 2012). Besides, there is no difference in terms of transplantation with undifferentiated ADSCs or differentiated ones in nerve regeneration (Orbay et al., 2012), suggesting the treatment time can be shortened by direct ADSC transplantation.

It is known that hypoxic expansion decreases in senescence, increases in proliferation, and enhances multilineage differentiation potential in BMSCs (Grayson et al., 2006; Adesida et al., 2012). The purpose of this study was to examine whether hypoxic expansion could increase the neuronal differentiation potential *in vitro* and the treatment effect *in vivo* in ADSCs and compare the neuronal differentiation potential between ADSCs and BMSCs following hypoxic expansions.

## Materials and Methods

### Isolation of Human BMSCs and ADSCs

The human BMSC cell line was obtained from Professor Shih-Chieh Hung’s Lab (Lin et al., 2015). Fat tissues were aspirated from healthy female donors undergoing liposuction of the abdomen. The procedures were approved by the Institutional Review Board (IRB) of the Taipei Veterans General Hospital. The fat tissue needed in this study was then stored at 4°C and the stromal-vascular fraction (SVF), which contained human ADSCs, was separated within 24 h through SVF isolation as previously described (Yoshimura et al., 2006; Wu et al., 2018). The excised fat tissue was carefully cut into small uniform pieces to yield similar digestion efficiency. The oil layer on the top and the aqueous layer were removed by centrifugation, and the collected fat volume was recorded. Excess blood was removed from the SVF after having been washed two times with cold PBS or the Hank’s Balanced Salt Solution (HBSS; Gibco, Carlsbad, CA, United States). 0.075% (w/v) collagenase (Sigma-Aldrich, St. Louis, MO) in a prewarmed HBSS buffer was prepared for one to twofold fat volume and filtered with a 0.22-μm filter. The mix ratio of the collagenase buffer and fat was 1:1 or 2:1. After having been mixed, the fat mixture was incubated in a shaking water bath at 37°C for 30 min. Following digestion, a half volume of the Dulbecco’s Modified Eagle Medium (DMEM; Gibco) and 10% fetal bovine serum (FBS; Invitrogen, Carlsbad, CA) were thoroughly mixed with the fat mixture for neutralization, which then underwent centrifugation and was washed with PBS three times to remove the collagenase. The pellets contained SVF and some fibrotic tissue suspended with an appropriate amount of the medium for cell counting afterward. Series of filtrations were performed with 100, 70 (optional), and 40-μm filters and filtered. Following isolation, 4–7 × 10^5^ living cells in 1 ml of the normal human fat tissue could be extracted.

### Cell Expansion

For the cell culture, approximately 2–3 × 10^6^ cells at passages 3–6 were seeded in one 10-cm dish and cultured in a DMEM containing 10% FBS and Antibiotic-Antimitotic solution (Corning Life Science, New York, United States). For hypoxic expansion, the cells were cultured in 94% N_2_, 5% CO_2_ and 1% O_2_ (Yew et al., 2012). For the normoxic culture, the cells were cultured in 5% CO_2_ and 20% O_2_ (a Forma Series II Water Jacketed CO_2_ incubator, Thermo Fisher Scientific Inc., Waltham, MA, United States).

### Neuronal Differentiation

The neuronal differentiation procedure was performed according to a previous study (Hung et al., 2002). Briefly, the cells with 80% confluence were induced by a DMEM supplemented with 50 μM All-trans retinoic acid (RA; Sigma-Aldrich) and 1 mM 2-Mercaptoethanol (β-ME; Sigma-Aldrich), which were placed in a 37°C, 5% CO_2_ and 20% O_2_ incubator for 24 hr. Then the cells were maintained with DMEM solution containing 1% FBS for 7 days.

### Real-Time Quantitative Reverse Transcription Polymerase Chain Reaction (RT-qPCR) Analysis

Total RNA was extracted from 1 × 10^6^ cells using the Trizol reagent (Invitrogen, Carlsbad, CA) and adjusted to 1,000 ng. Random sequence primers were used to prime reverse transcription reactions, and the first strand complementary DNA (cDNA) synthesis was achieved by employing the iScript^TM^ cDNA Synthesis Kit (Bio-Rad^®^, Hercules, CA). cDNA, which was synthesized by the M-MuLV reverse transcriptase, was applied as the template to perform the PCR reaction in a 20-μl reaction mixture with specific primers. The sequences of the primers in this study were listed in Table 1. A Fast SYBR^®^ Green Master Mix (Applied Biosystems, Foster City, CA) was used to perform Real-time qPCR, with 40 cycles consisting of repeating the following steps: the holding stage for 10 min at 95°C, denaturation for 3 s at 95°C, and annealing stage for 30 s at 60°C. The glyceraldehyde-3-phosphate dehydrogenase (*GAPDH*) gene was detected as an internal control to confirm the efficiency of the PCR and cDNA synthesis. The results were produced using the comparative C_*T*_ (ΔΔC_*T*_) method.

**TABLE 1:** Primer sequences of the neuron markers used for RT-qPCR analysis.

Gene	Accession number	Forward primer (5′–3′)	Reverse primer (5′–3′)
*TUBB3*	NM_139254	GCCAAGTTCTGGGAGGTCATC	GTAGTAGACACTGATGCGTTCCA
*MAP2*	X54100.1	CAAACGTCATTACTTTACAACTTGA	CAGCTGCCTCTGTGAGTGGAG
*NEFH*	NM_012607	AGTGGTTCCGAGTGAGATTG	CTGCTGAATTGCATCCTGGT
*NEFM*	NM_017029	AGTGGTTCAAATGCCGCTAC	TTTTCCAGCTGCTGGATGGT
*CD90*	NM_006288	ATCGCTCTCCTGCTAACAGTC	CTCGTACTGGATGGGTGAACT
*CD105*	NM_001114753	TGCACTTGGCCTACAATTCCA	AGCTGCCCACTCAAGGATCT
*GAPDH*	NM_017008	CAACTCCCTCAAGATTGTCAGCAA	GGCATGGACTGTGGTCATGA

### Immunofluorescence (IF) Staining and Quantification

Primary antibodies for IF staining against Class III β-tubulin (TUBB3; Santa Cruz, CA, United States), microtubule-associated protein 2 (MAP2; GeneTex Inc., Irvine, CA), neurofilament medium polypeptide (NEFM; OriGene Technologies, Rockville, MD), and neurofilament light polypeptide (NEFL; OriGene Technologies, Rockville, MD) were added on 7 days differentiated cell slides at suitable dilution. Secondary antibodies with green fluorescence protein against the primary antibodies were used for fluorescence analysis. 4,6-diamidino-2-phenylindole (DAPI; Sigma-Aldrich) for nuclear staining (blue) was used to counterstain the slides. The samples incubated with the secondary antibodies but without the first antibodies were employed as a negative control. Green fluorescence intensity was measured by the Image-Pro Plus (v4.5.0.29, Media Cybernetics, Silver Spring, MD). Total florescence intensity from 120 to 140 cells in six fields on each slide was measured, and the average intensity of individual cells was also calculated.

### Animal Model

The procedure of the collection of rat ADSCs was the same as that of the human’s. Each Sprague-Dawley rat (250–300 g in weight) was anesthetized with the Zoletil^®^ 50 (Virbac, Carros, France) for 1 ml/kg. A 5-mm segmental nerve defect in the right sciatic nerve of each of all the rats was exposed by a dorsal gluteal-splitting approach, with the contralateral leg similarly exposed, but not the nerve defect, used as a sham control (*n* = 24). Each defect area was connected with a 5 mm long (∼1.53 mm inner diameter) microporous polylactide conduit (Hsu et al., 2013) and sutured with four 8–0 nylon sutures through the epineurium. Before injections, the ADSCs were pre-incubated with superparamagnetic iron oxides (SPIOs) for histological iron staining. The matrigel matrix (Corning life science) was mixed with 1 × 10^6^ normoxic ADSC (*n* = 6), hypoxic ADSC (*n* = 6) or hypoxic BMSC (*n* = 6) cells or without cells (*n* = 3) in 100 μl volumes and injected into the conduits for nerve reconstruction, and followed by wound closures with postsurgical care. The sciatic nerve injury with a conduit connection but without a Matrigel injection was specified as the non-treatment group (*n* = 3). The experimental protocols and follow-up animal care were compliant with the institutional animal welfare guidelines of the Taipei Veterans General Hospital. The rats were anesthetized with 5 ml/kg Zoletil^®^ 50 (Virbac) and then transcardially perfused with 4% paraformaldehyde in 0.1M PBS (pH 7.4) after treatments for 6 weeks. The regenerated sciatic nerve was removed from each conduit and prepared for the follow-up analysis. All the procedures involving the animals were approved by the Institutional Animal Care and Use Committee of the Taipei Veterans General Hospital.

### Gastrocnemius Muscle Weight Analysis

The gastrocnemius muscles from the limbs of those without treatment, Matrigel, hypoxic, and normoxic ADSC treatments were dissected and immediately weighed, and then compared with the limbs of the sham group. The weights of the gastrocnemius muscles in each group that had been normalized with those of the sham group were converted to percentages as recovery rates, and the sham group was defined as 100% (Hsueh et al., 2014).

### Histological Stain and Myelinated Fiber Quantification

The middle parts of the regenerated sciatic nerves in the conduits were cross sectioned for histological analysis; the paraffin-embedded nerve tissue sections were deparaffinized in xylene. Rehydration was performed with 100 to 70% graded alcohol, and the organic solution was removed by rinsing in ddH_2_O. Hematoxylin and eosin (HE) staining (Sigma-Aldrich), toluidine blue staining (Sigma-Aldrich) (Ebrahimi et al., 2018), myelin basic protein (MBP) immunostaining (Merolli et al., 2019) and iron staining (Sigma-Aldrich) (Liu and Ho, 2017) were used to stain the sections, with the results being examined by a light microscope AX80 (Olympus, Tokyo, Japan).

Photographs of the toluidine blue staining sections taken under light microscopy at a 400 × magnification were used for quantification. The total numbers of the myelinated fibers in each section from three regenerated nerves were counted and normalized with the whole background of each section using the Image-Pro Plus (v4.5.0.29, Media Cybernetics) (Chen et al., 2020). The results showed the mean values calculated from three sections in each group.

### Statistical Analysis

A quantitative analysis of the results was undertaken using the Prism (version 5.03, GraphPad, La Jolla, CA, United States). The Mann-Whitney *U*-test was used for comparing the two groups, and one-way analysis of variance (ANOVA) was for multi-group comparison. The results were presented as means ± standard errors; a *p*-value less than 0.05 was considered significant.

## Results

### Neuronal Differentiation of Normoxic and Hypoxic ADSCs *in vitro*

**FIGURE 1:**
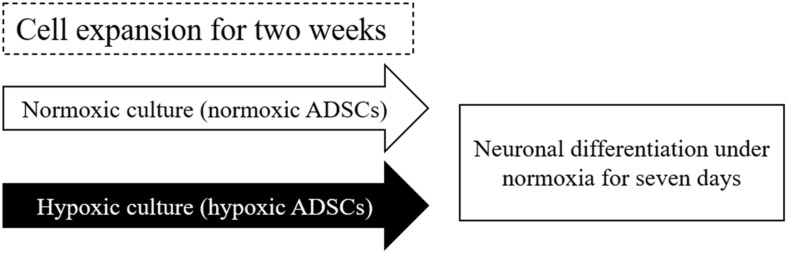
Scheme of normoxic and hypoxic expansion and neuronal differentiation. Freeze-thaw or continuous-culturing human adipose-derived stem cells (ADSCs) at passages 3–5 were cultured under normoxic (normoxic ADSCs) or hypoxic condition (hypoxic ADSCs) for 2 weeks. After expansion, both the normoxic and hypoxic ADSCs were exposed to 2-Mercaptoethanol (β-ME) to induce neuronal differentiation for 7 days. The neuron-related genes and proteins of the normoxic and hypoxic ADSC differentiated cells were monitored from days 0 to 7.

**FIGURE 2:**
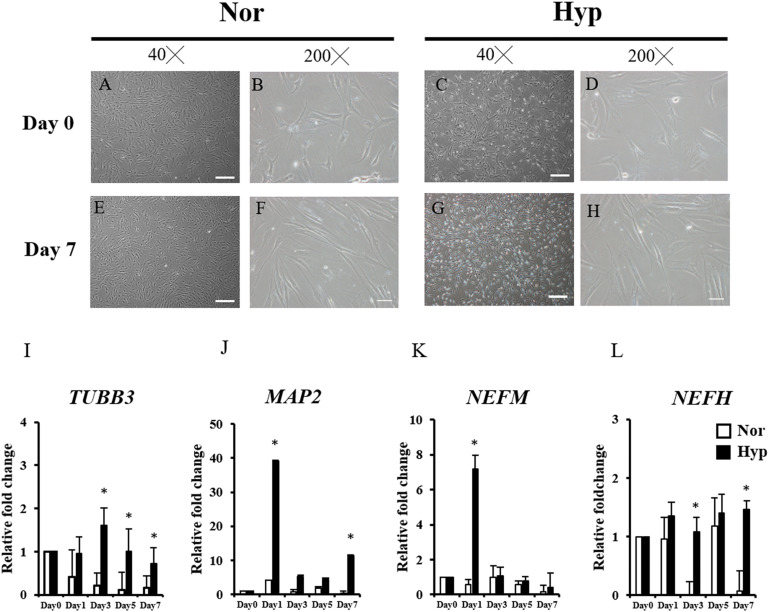
Morphology and neuron-associated gene expressions of ADSC differentiated neuron-like cells. The morphologies of undifferentiated ADSCs expanded in normoxia **(A,B)** and hypoxia **(C,D)** before induction (Day 0) were examined using phase-contrast microscopy. The neuron-like morphologies differentiated from the normoxic **(E,F)** hypoxic ADSCs **(G,H)** under normoxia were observed on day 7 by β-ME induction. (Magnification × 40; the scale bar = 200 μm; magnification × 200; the scale bar = 50 μm). Genes including Class III β-tubulin (*TUBB3*) **(I)**, microtubule-associated protein 2 (*MAP2*) **(J)**, neurofilament medium polypeptide (*NEFM*) **(K)**, and neurofilament heavy polypeptide (*NEFH*) **(L)** of the normoxic and hypoxic ADSC differentiated cells were detected by RT-qPCR from days 0 to 7, and all the gene expression values were normalized to the expression of glyceraldehyde-3-phosphate dehydrogenase (*GAPDH*). The values from day 1 to day 7 were compared with those of day 0 to show the relative fold change. The values were presented as mean ± SD with three replicates of the experiments. Statistical significances between the two groups were analyzed using the Mann-Whitney *U*-test. “^∗^” represented *P* < 0.05.

After 2 weeks of expansion under normoxic (normoxic ADSCs) or hypoxic (hypoxic ADSCs) conditions, the ADSCs proceeded to neuronal differentiation in normoxia for 7 days. The process was showed in Figure 1. The undifferentiated ADSCs on day 0 were imaged using phase-contrast microscopy (Figures 2A–D). The neuron-like morphologies showing stretches on differentiated normoxic (Figures 2E,F) and hypoxic ADSCs (Figures 2G,H) with β-ME induction were examined on day 7, which confirmed that both normoxic and hypoxic ADSCs could be induced to experience neuronal differentiation. RT-qPCR was used to confirm the neuronal markers of the normoxic and hypoxic ADSC differentiated neuron-like cells by monitoring the gene expressions of *TUBB3* (Figure 2I), *MAP2* (Figure 2J), *NEFM* (Figure 2K), and *NEFH* (Figure 2L) through the time course. The *TUBB3* expressions were significantly increased in the hypoxic ADSCs compared with those of the normoxic ones after 3 days of induction (*P* < 0.05). The expressions of *MAP2* were highly induced in the hypoxic ADSC group on days 1 and 7 of differentiation (*p* < 0.05). The *NEFM* gene were highly expressed in the hypoxic ADSC differentiated neuron-like cells on day 1 (*P* < 0.05), and *NEFH* was also significantly enhanced on days 3 and 7 (*P* < 0.05). In addition, the differentiation efficiency was determined by detecting the cell marker genes of ADSCs, such as CD90 and CD105, after differentiation. RT-qPCR detection of marker gene expressions in the normoxic or hypoxic ADSCs on day 0 and differentiated neuron-like cells on day 7 was measured and compared. The result showed that there were no significant differences in the ADSC markers of the normoxic and hypoxic ADSC differentiated neuron-like cells on day 7 (Supplementary Figure 1).

**FIGURE 3:**
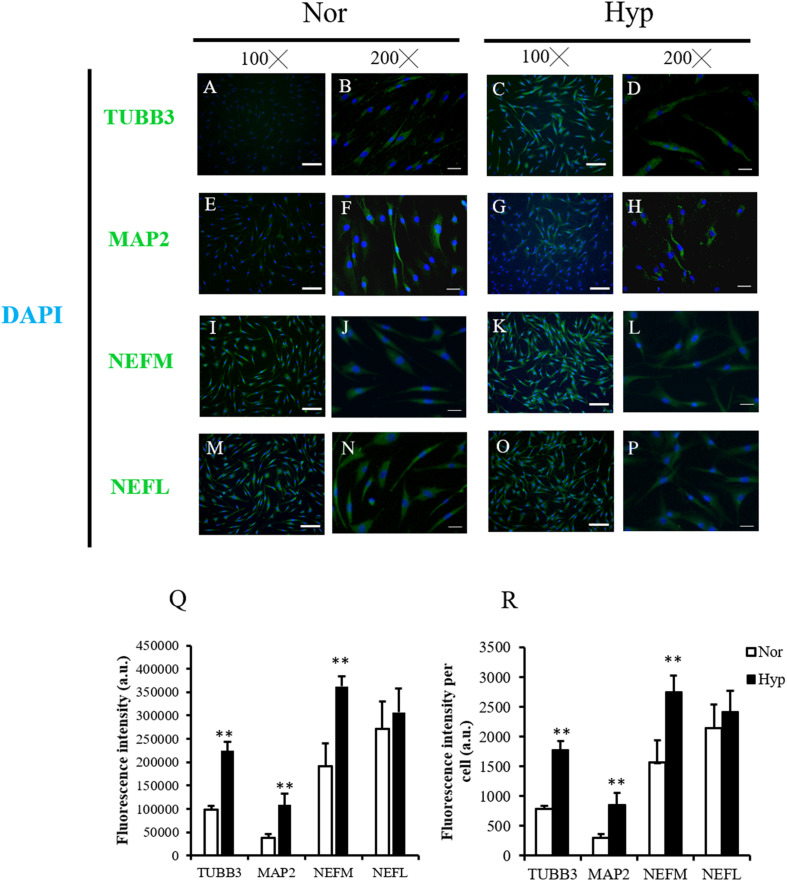
Neuron marker detection of the normoxic and hypoxic ADSC differentiated neuron-like cells. **(A–D)** Class III β-tubulin (TUBB3), **(E–H)** microtubule-associated protein 2 (MAP2), **(I–L)** neurofilament medium polypeptide (NEFM), and **(M–P)** neurofilament Light polypeptide (NEFL) of the normoxic and hypoxic ADSC differentiated neural-like cells on day 7 of differentiation were stained by immunofluorescence staining (Magnification × 100; the scale bar = 100 μm; magnification × 200; the scale bar = 50 μm). **(Q)** Total immunofluorescence intensities of 120–150 cells from six fields were quantified by the Image-Pro Plus v4.5.0.29. The intensity of each cell was also calculated **(R)**. Values are presented as mean ± SD with three replicates of the experiments. Statistical significances between the two groups were analyzed using the Mann-Whitney *U*-test. “^∗∗^” represented *P* < 0.01.

IF staining was used to confirm the neuronal markers of the normoxic ADSC or hypoxic ADSC differentiated neuron-like cells on day 7 by examining the expressions of the TUBB3 (Figures 3A–D), MAP2 (Figures 3E–H), NEFM (Figures 3I–L), and NEFL (Figures 3M–P) proteins. The total fluorescence intensity and average fluorescence intensity of the TUBB3, MAP2, and NEFM protein expressions in the hypoxic ADSCs were significantly higher than those of the normoxic ADSCs (*p* < 0.01, Figures 3Q,R). NEFL protein was also higher in the hypoxic ADSC group although the result was not significantly different. The aforementioned results implied hypoxic expansion prior to differentiation might preserve neuronal differentiation potential in ADSCs.

### ADSC Transplantation in the Animal Model

**FIGURE 4:**
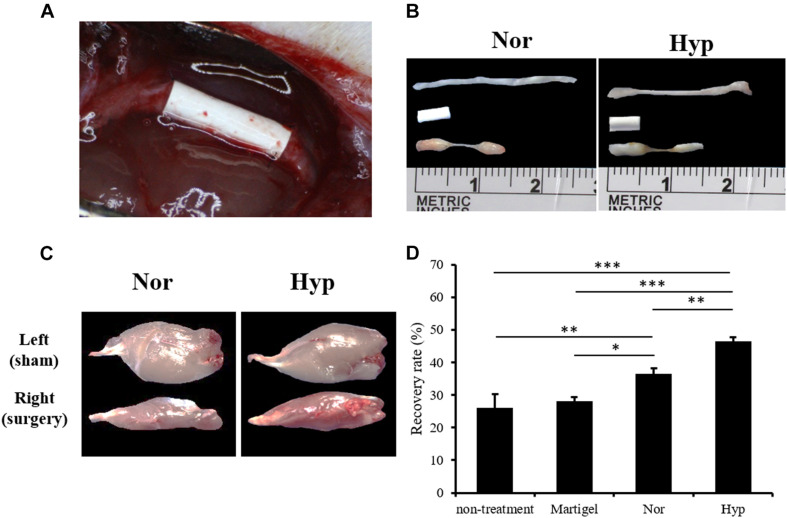
The animal model of the sciatic nerve defect and outcome analysis of the ADSC treatment. **(A)** A polylactide conduit connecting a 5-mm sciatic nerve defect by surgical implantation in the Sprague-Dawley rats. The animals were sacrificed after 6 weeks. **(B)** The dissected normal sciatic nerve, implanted conduit, and regenerated nerve from the normoxic, or hypoxic ADSCs were exhibited from the top to the bottom, respectively. **(C)** The dissected gastrocnemius muscles from the left limbs of the rats as the sham group were showed in the upper panel. The lower panel displayed the atrophied muscles from the right limb with sciatica receiving a treatment of the normoxic or hypoxic ADSCs in the conduits. **(D)** The weights of the gastrocnemius muscles were measured and normalized with those of the sham group to calculate the recovery rate. The values were presented as mean ± SD with three replicates of the experiments. Statistical significance between the multiple groups was analyzed using the one-way analysis of variance (ANOVA) analysis. “^∗^” represented *p* < 0.05, “^∗∗^” represented *p* < 0.01, and “^∗∗∗^” represented *p* < 0.001.

The surgical conduit connecting the distal stump of the rat sciatic nerve defect was imaged in Figure 4A and the middle of Figure 4B. The normoxic or hypoxic ADSCs mixed with Matrigel were injected into the conduit. The intact sciatic nerve was dissected and exhibited in the upper part of Figure 4B, and the regenerated nerve fibers in the conduits filled with the normoxic or hypoxic ADSCs injections were shown at the bottom of Figure 4B. The ankle of the rat limb with the sciatic nerve incision without treatment was not straightened while the feet were pulled, which was called an ankle contracture, and the gastrocnemius muscle would shrink. Nevertheless, the atrophied gastrocnemius muscle would repair as long as the nerves were regenerated (Figure 4C). The recovery rates which were calculated according to the weights of the gastrocnemius muscle in the hypoxic ADSCs (mean = 46% ± 1.39) were significantly higher than those of the normoxic group (mean = 36% ± 1.79, *p* < 0.01), non-treatment group (mean = 25% ± 4.2, *p* < 0.001), and the Matrigel group (mean = 28% ± 1.17, *p* < 0.001) (Figure 4D).

**FIGURE 5:**
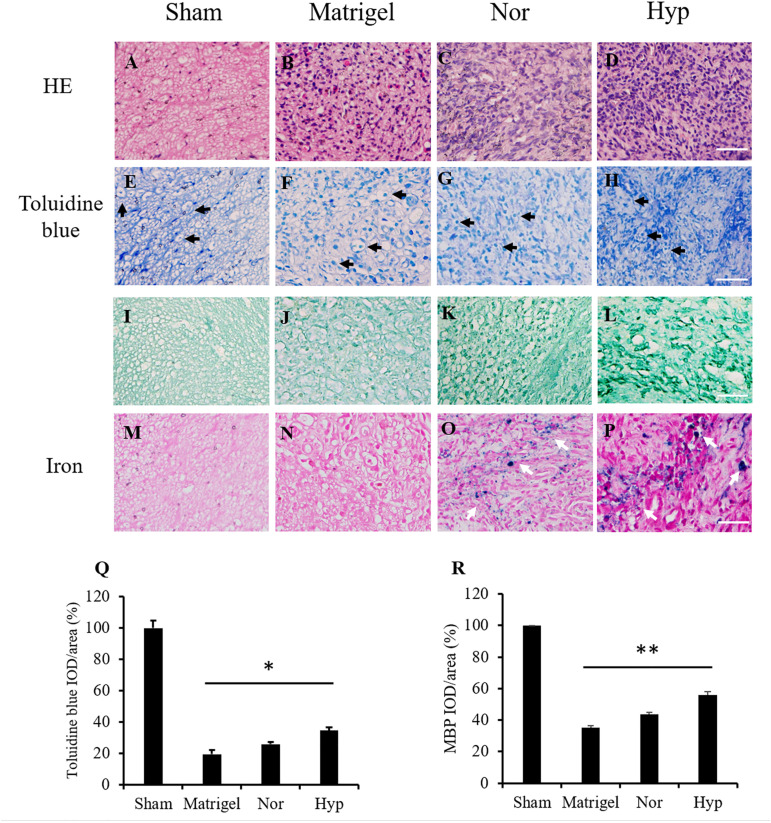
Histologic staining of the nerve sections. The sections in the middle part of the sciatic nerve from the sham group **(A)** and the regenerated nerve from the Matrigel **(B)**, normoxic **(C)**, and hypoxic rat ADSCs (rADSCs) **(D)** were stained by hematoxylin and eosin (HE) staining. Toluidine blue was applied to detect nerve fibers (black arrows) from the sham **(E)**, Matrigel **(F)**, normoxic, **(G)** and hypoxic rADSCs **(H)**, the results of which were showed in the second row. Myelin basic protein (MBP) immunostaining was applied to detect myelin in the sham **(I)**, Matrigel **(J)**, normoxic **(K)**, and hypoxic rADSCs **(L)**, the results of which were showed in the third row. Iron particles (white arrows) in the injected rADSCs were also tracked in the sham **(M)**, Matrigel **(N)**, normoxic **(O)** and hypoxic rADSCs **(P)** (Magnification × 400; the scale bar = 100 μm). The nerve fibers from **(E–H)** and **(I–L)** were quantified by the Image-Pro Plus (v4.5.0.29, Media Cybernetics), respectively. **(Q,R)** The coverage percentages of the myelinated nerve fibers from three sections of each group **(E–H)** or **(I–L)** were calculated and normalized with those of the sham group. The values were converted to percentages and expressed as mean ± SD with three experimental replicates. Statistical significance between the multiple groups was analyzed using the one-way analysis of variance (ANOVA) analysis. “*” represented *P* < 0.05, “**” represented *P* < 0.01.

The section structure of the normal sciatic nerve and regenerated nerves in the conduits from the Matrigel, and with normoxic or hypoxic ADSC treatments were stained by HE staining (Figures 5A–D) to present the structures of the nerve section. Toluidine blue staining and MBP immunostaining were used to detect and quantify the regenerated myelinated nerve fibers (Figures 5E–L). The integrated optical density (IOD) of the stained fibers were normalized with those of the sham group and converted to percentages. The mean percentage value of the sham group was defined as 100%. The mean percentage of the nerve fiber detected by toluidine blue staining in the hypoxic ADSC group was 34%, which was significantly enhanced compared with that of the Matrigel group (19%, *p* < 0.05), and also higher than that of the normoxic ADSCs group (25%) (Figure 5Q). In addition, the mean percentage of the myelinated nerve fiber detected by MBP immunostaining in the hypoxic ADSC group was 55.9%, which was also significantly higher than that of the Matrigel group (35.12%, *p* < 0.01), and also higher than that of the normoxic ADSC group (43.6%) (Figure 5R).

To detect the SPIO marked ADSCs in the regenerated nerve fibers, the nerve section of the sham group was referred to as a control (Figure 5M). There was no detection of iron marks in the regenerated nerves of the Matrigel group grown from the host stump nerve (Figure 5N). The SPIO previously seeded in the ADSCs was tracked by iron staining (Figures 5O,P).

### Comparisons of the Neuronal Differentiation of Hypoxic ADSCs and Hypoxic BMSCs

To compare the potential of neuronal differentiation in the hypoxic ADSCs and BMSCs following hypoxic expansion (hypoxic BMSCs), neuron-associated genes, including *TUBB3* and *MAP2* (Supplementary Figures 2A,B), were monitored through the time course by RT-qPCR. The expressions of *MAP2* in the hypoxic ADSC differentiated neuron-like cells were significantly higher than those of the hypoxic BMSCs on days 1 and 7. The sciatic nerve injury rat model was also used to compare the outcomes of the hypoxic ADSC and hypoxic BMSC treatment. The recovery of the atrophied gastrocnemius muscle in these two groups was similar since the mean value of the hypoxic ADSCs was 49%, and that of the hypoxic BMSCs was 45% (Supplementary Figures 2C,D).

## Discussion

A comparison of ADSCs and BMSCs in terms of peripheral nerve regeneration on sciatic nerve injury was examined, and the study showed no significant difference in the results of ADSC and BMSC treatments for sciatic nerve function recovery (Fernandes et al., 2018). In this study, the neuronal differentiation potential of ADSCs and BMSCs expanded under hypoxia was also compared. The results showed the neuron markers were enhanced in the hypoxic ADSCs while neuronal differentiation proceeded *in vitro*. However, the results of the recovered gastrocnemius muscles of both cell treatments were similar, which demonstrated the neuron repair function of ADSCs and BMSCs were comparable. Moreover, the application of ADSCs was more convenient compared with that of BMSCs due to the fact that the collection of ADSCs was more advantageous, and there was a greater gain in cell numbers, better proliferation, and less effects of age (Chen et al., 2012).

The physiological oxygen tension in the original niches of BMSCs and ADSCs is relatively lower than the ambient air. The hypoxic condition having oxygen tension of around 1–7% in bone marrow (Eliasson and Jonsson, 2010; Spencer et al., 2014) supports BMSCs in preserving their biological characters (Mohyeldin et al., 2010; Abdollahi et al., 2011), enhances proliferation (Adesida et al., 2012), and reduces senescence (Tsai et al., 2011). The oxygen concentration in adipose tissue varies from 3 to 11%, depending on the blood flow (Goossens and Blaak, 2012). A hypoxic culture is also found to reduce ADSC senescence, increase proliferation, and retain the differentiation properties (Weijers et al., 2011; Valorani et al., 2012). Hypoxia is considered an important condition to preserve the function of stem cells (Mohyeldin et al., 2010). The surgical nerve conduits without blood vessels are also a hypoxic microenvironment that would make macrophages activate the hypoxia-inducible factor (HIF-1α) to secrete VEGF-A and vascularization. The newborn blood vessels resolve the hypoxia in the conduit and lead the Schwann cell to pass the lesion (Cattin et al., 2015). Thus, BMSCs or ADSCs that continue culturing under hypoxia *in vitro* would survive in the hypoxic environment of the conduit before new blood vessels are formed. Furthermore, ADSCs would secrete VEGF, BDNF, NGF, and GDNF that enhance host SCs to undergo intrinsic healing (Faroni et al., 2014).

BMSCs continue culturing under hypoxia and are proven to reduce cellular senescence (Tsai et al., 2011). Since an ADSC culture in hypoxic conditions has been demonstrated to enhance proliferation and preserve stemness (Fotia et al., 2015), the hypoxia precondition for 24 h before transplantation was also found to benefit ADSCs in improving angiogenesis and neuroprotection and further enhance the therapeutic potential for erectile dysfunction (Wang et al., 2015). In this study, the ADSCs that were expanded and kept culturing under hypoxia for three to five passages before the normoxic neuronal differentiation *in vitro* or transplantation *in vivo* could preserve the potential of neuronal differentiation.

The limitations of this study are as follows. The process of ADSC neuronal differentiation *in vitro* was examined for a maximum of 7 days, and the mature ADSC neuron cells were not observed. The differentiation efficiency which had been determined by detecting the ADSC marker genes also pointed out the limitation of the induction time. The ADSC markers could still be detected after the 7 days induction. Although the neuron-like cells differentiated from the ADSCs expressed a neuron marker, this did not guarantee the cells would grow into functional neurons *in vitro*. In addition, differentiated or undifferentiated ADSC transplantation displayed no difference in nerve regeneration (Orbay et al., 2012). In this study, the ADSCs were transplanted directly without neuronal differentiation, revealing the beneficial therapeutic effects of ADSCs might not be due to the neuronal differentiation function but the secretion of growth factors (Sowa et al., 2012). The neuron in the regenerated nerve fiber could not be confirmed to be developed by ADSCs. As to the limitations of the animal model experiment, animal behavior tests such as the rotarod test, functional assessment, and nerve conduction velocity (NCV) were not conducted in this study. Moreover, a nerve defect of humans is much longer than that of an experimental rat, which might be the reason the result did not show many differences.

## Conclusion

ADSCs expanded under both normoxic or hypoxic conditions all make neuronal differentiation occur in normoxia. This study suggested that hypoxic expansion enhanced the neuronal differentiation potential of ADSCs *in vitro* and *in vivo*. ADSC expansion under hypoxia before transplantation may be a beneficial step to improve the clinical application of ADSCs in nerve regeneration and nerve repair, which could be administered to treat other peripheral neuropathy conditions.

## Data Availability Statement

The original contributions presented in the study are included in the article/Supplementary Materials, further inquiries can be directed to the corresponding author/s.

## Ethics Statement

The studies involving human participants were reviewed and approved by the Institutional Review Board (IRB) of the Taipei Veterans General Hospital. The patients/participants provided their written informed consent to participate in this study. The animal study was reviewed and approved by the Institutional Animal Care and Use Committee of the Taipei Veterans General Hospital.

## Author Contributions

S-HW, Y-TL, and J-PW contributed to conception and design, acquisition of data, analysis, and interpretation of data. All authors participated in drafting the article or revising it critically for important intellectual content and gave final approval of the version to be submitted and any revised version.

## Conflict of Interest

The authors declare that the research was conducted in the absence of any commercial or financial relationships that could be construed as a potential conflict of interest.

## Abbreviations

ADSCs: Adipose-derived mesenchymal stem cells
BMSCs: Bone marrow-derived mesenchymal stem cells
SCs: Schwann cells
VEGFs: Vascular endothelial growth factors
BDNFs: Brain-derived growth factors
NGFs: Nerve growth factors
GDNFs: Glial line-derived neurotrophic factors
IRB: Institutional Review Board
SVF: Stromal-vascular fraction
PBS: Phosphate-buffered saline
HBSS: Hank’s Balanced Salt Solution
DMEM: Dulbecco’s Modified Eagle Medium
FBS: fetal bovine serum
β-ME: 2-Mercaptoethanol
RT-qPCR: quantitative reverse transcription polymerase chain reaction
GAPDH: Glyceraldehyde-3-phosphate dehydrogenase
IF: Immunofluorescence
TUBB3: Class III β-tubulin
MAP2: Microtubule-associated protein 2
NEFM: neurofilament medium polypeptide
NEFL: neurofilament light polypeptide
NEFH: neurofilament heavy polypeptide
cDNA: complementary DNA
MBP: myelin basic protein
IOD: integrated optical density
DAPI: 4,6-diamidino-2-phenylindole
SPIO: Superparamagnetic iron oxides
HE: Hematoxylin and eosin
ANOVA: One-way analysis of variance
HIF-1 α: Hypoxia-inducible factor-1 α
NCV: Nerve conduction velocity.
