# Supplementary material for: Adipose-Derived Mesenchymal Stem Cells From a Hypoxic Culture Improve Neuronal Differentiation and Nerve Repair
Source: Front Cell Dev Biol. 2021 Apr 30;9:658099. doi: 10.3389/fcell.2021.658099 (PMC8120285; doi:10.3389/fcell.2021.658099)
Supplement: Supplementary file 1 [file Presentation_1.pdf]

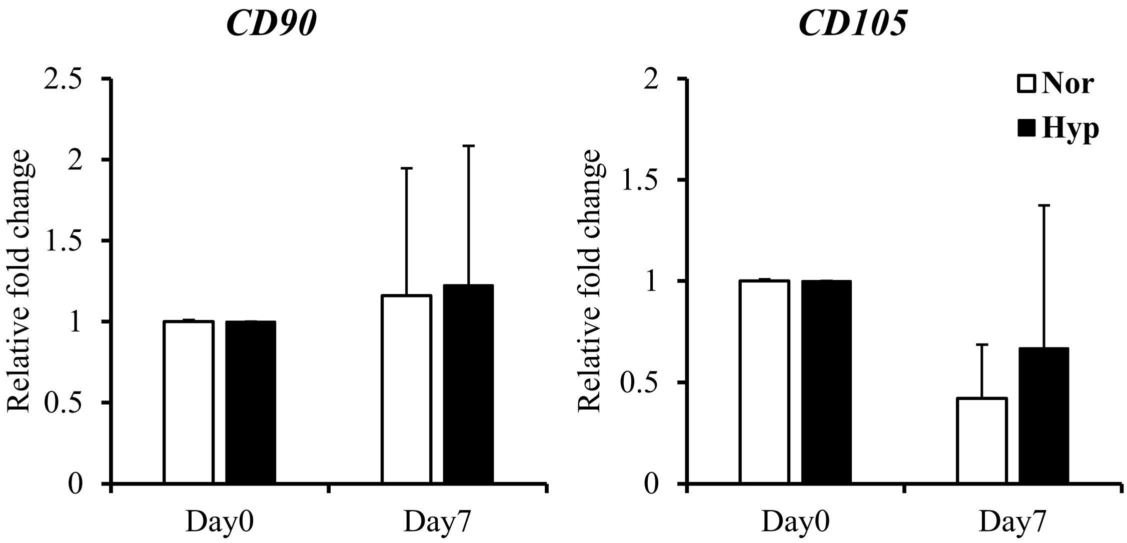

Supplementary figure S1. Comparison of the normoxic and hypoxic ADSCs for differentiation efficiency. The cell marker genes of ADSCs, including CD90 and CD105 in the normoxic or hypoxic ADSCs on day 0 and in differentiated cells after 7 days of neuronal differentiation were detected using RT-qPCR. The gene expression values were normalized to the expression of glyceraldehyde-3-phosphate dehydrogenase (*GAPDH*). The values were presented as mean ± SD with three replicates of the experiments. Statistical significances between the two groups were analyzed using the Mann-Whitney U test. There were no significant differences between the values of Day0 and Day7.

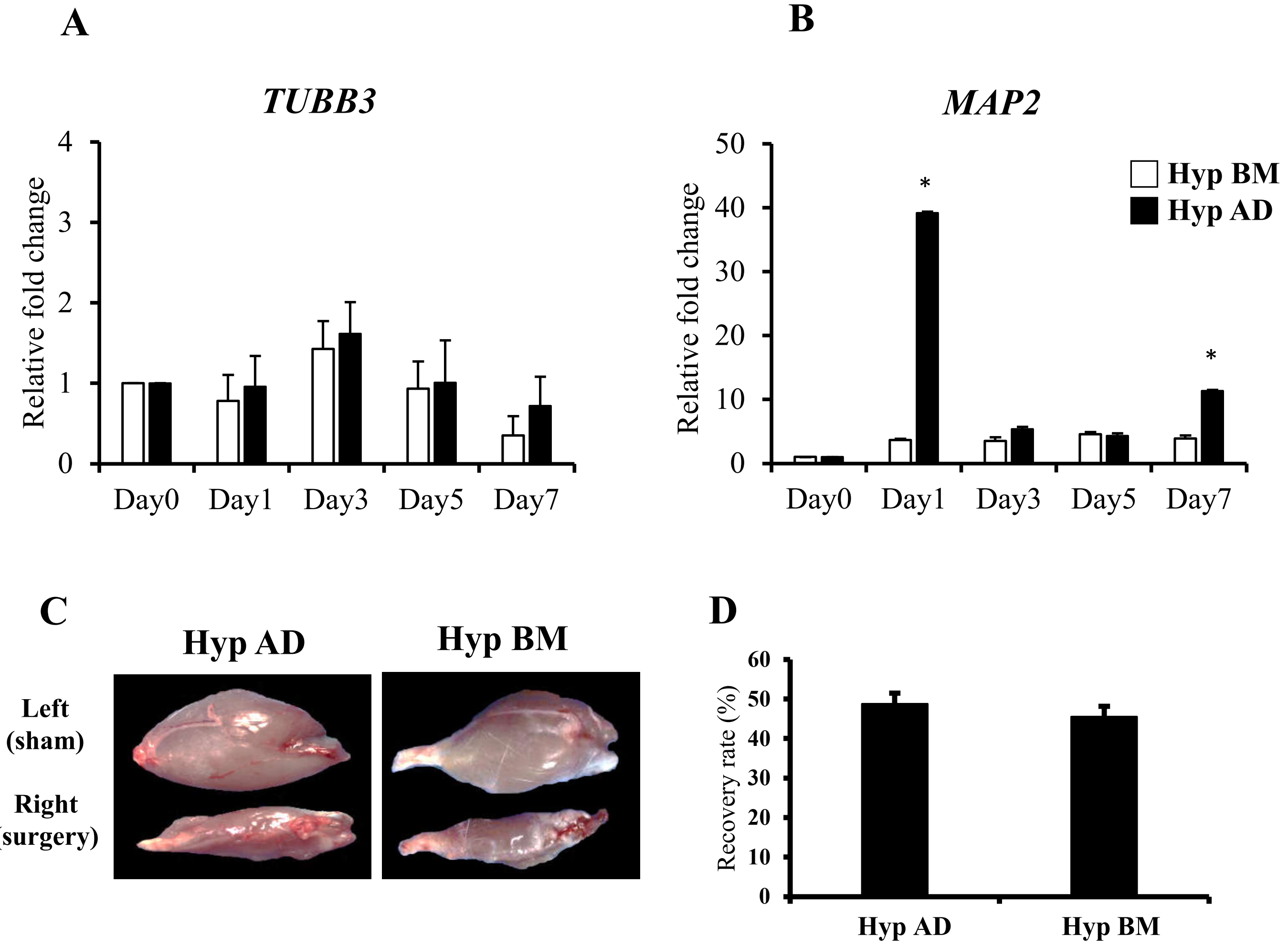

Supplementary figure S2. Comparison of the hypoxic ADSCs and hypoxic BMSCs for peripheral nerve regeneration potential. The expressions of genes, including *TUBB3* (A) and *MAP2* (B), of the hypoxic ADSC (Hyp AD) and BMSC (Hyp BM) differentiated cells were compared and detected by RT-qPCR from day 0 to day 7, and all the gene expression values were normalized to the expression of *GAPDH*. The values were presented as mean ± SD with three replicates of the experiments. (C) The dissected gastrocnemius muscles from the left limbs of the rats as the sham group were shown in the upper panel. The lower panel exhibited the atrophied muscles from the right limbs with sciatica receiving a treatment of the hypoxic ADSCs or BMSCs in the conduits. (D) The weights of the gastrocnemius muscles were measured and normalized with those of the sham group to calculate the percentages of the recovery rates. Statistical significance between the two groups was analyzed using the Mann-Whitney U test. There was no significant difference between groups.
